# Effects of Poloxamer Content and Storage Time of Biodegradable Starch-Chitosan Films on Its Thermal, Structural, Mechanical, and Morphological Properties

**DOI:** 10.3390/polym13142341

**Published:** 2021-07-17

**Authors:** Abril Fonseca-García, Carolina Caicedo, Enrique Javier Jiménez-Regalado, Graciela Morales, Rocio Yaneli Aguirre-Loredo

**Affiliations:** 1 Centro de Investigación en Química Aplicada (CIQA), Blvd. Enrique Reyna Hermosillo 140, Saltillo, Coahuila 25294, Mexico; abril.fonseca@ciqa.edu.mx (A.F.-G.); enrique.jimenez@ciqa.edu.mx (E.J.J.-R.); graciela.morales@ciqa.edu.mx (G.M.); 2 CONACYT-CIQA, Blvd. Enrique Reyna Hermosillo 140, Saltillo, Coahuila 25294, Mexico; 3 Grupo de Investigación en Química y Biotecnología (QUIBIO), Facultad de Ciencias Básicas, Universidad Santiago de Cali, Pampalinda, Santiago de Cali 760035, Colombia; carolina.caicedo03@usc.edu.co

**Keywords:** biodegradable packaging, biopolymer, starch, storage conditions, thermal properties, physicochemical properties

## Abstract

Biodegradable packaging prepared from starch is an alternative to fossil-based plastic packaging. However, the properties of starch packaging do not comply with the necessary physicochemical properties to preserve food. Hence, in a previous study, we reported the preparation of a composite polymer material based on starch-chitosan-pluronic F127 that was found to be an adequate alternative packaging material. In this study, we modified the physicochemical properties of this material by storing it for 16 months under ambient conditions. The results indicate that the incorporation of pluronic F127 in the blend polymer can help avoid the retrogradation of starch. Moreover, at higher concentrations of pluronic F127, wettability is reduced. Finally, after storage, the materials exhibited surface modification, which is related to a color change and an increase in solubility, as well as a slight increase in stiffness.

## 1. Introduction

Synthetic-origin plastics have shaped various areas of our life, bringing with them numerous new and improved applications. However, their advantages, the environmental pollution resulting from their excessive use, slight or prolonged degradation, and inadequate waste disposal are critical. Studies have been focused on the development of polymeric materials of biological and biodegradable origin using biopolymers such as starch, cellulose, chitosan, and proteins that do not contribute to the accumulation of CO_2_ at the end of their life cycles but instead promote sustainable development in the face of any emerging ecological crisis [1,2,3,4].

Starch is an excellent choice for developing biodegradable packaging materials, owing to its abundance in nature and higher economical viability. As a nontoxic polymer, it can be safely used for food packaging applications; moreover, it does not impart flavor to the packaged food. Therefore, there is no risk of affecting the organoleptic properties of the packed foods [5].

However, biodegradable packaging based on starch and other biopolymers, such as chitosan or gelatin, are susceptible to hydration and thus do not resist high relative humidity conditions, direct contact with liquid water, or foods with high moisture content [6,7,8]. Therefore, through previous research, mixtures of starch with other biopolymers have been developed with better barrier properties against moisture [9,10,11,12]. A mixture of cornstarch, chitosan, and different proportions of a poloxamer, known as pluronic F127, was developed [11]. The study evaluated the effects of the pluronic concentration and its mechanical and thermal properties, permeability to water vapor, and solubility in water of its starch-chitosan films. The presence of the poloxamer considerably improved the moisture resistance of biodegradable packaging materials when made using starch in a concentration range of 1−5%. The packaging materials are less susceptible to water with a higher the concentration of poloxamer [11].

Storage time and conditions also significantly affect the properties of starch-based packaging materials due to the retrogradation exhibited by starch. Starch is composed of amylose and amylopectin. Amylose is a molecule with a mainly linear structure, whereas amylopectin is a highly branched molecule. The amylose-to-amylopectin ratio in corn starch granules is reported to be approximately 25/75 [13]. The proportion, size, and molecular organization of amylose and amylopectin, as well as the concentration of solids, significantly affects the retrogradation rate of materials made using starch [14,15]. The retrogradation of native starch mainly occurs due to the rearrangement of the amylose chains [16]. After storing for a long time, the amorphous molecules of the gelatinized starch tend to recrystallize to again form the ordered structures of double-helix crystallites. Thus, in gelatinized starch, both components, amylose and amylopectin, crystallize to form double helices generated from the external branches of amylopectin or amylose molecules [14,17].

The results obtained in a previous study were promising, suggesting this material, made using starch-chitosan-poloxamer, to be a viable alternative for producing packaging materials [11]. However, determining the behavior of this material during storage period is important.

This study hypothesized that new poloxamer-containing starch-chitosan formulations could maintain their structural integrity for several months, significantly delaying the starch retrogradation process that typically occurs in short periods. Therefore, in this study, the thermal, structural, mechanical, and morphological behaviors of corn starch-chitosan and poloxamer F127 stored for zero and sixteen months at room temperature were evaluated using differential scanning calorimetry (DSC), X-ray, FTIR-attenuated total reflection (ATR), tensile stress, wettability by the contact angle, color, and scanning electron microscopy (SEM) techniques.

## 2. Materials and Methods

### 2.1. Materials

The following biopolymers were used: corn starch from KMC (30% amylose; Brande, Denmark); shrimp shell chitosan (practical grade, deacetylation degree ≥ 75%; Sigma-Aldrich, Saint Louis, MO, USA); pluronic F127 (70% ethylene oxide; Sigma-Aldrich, Saint Louis, MO, USA). Glacial acetic acid (Productos Químicos Monterrey SA, Monterrey, Mexico) and glycerol (reagent grade; Meyer, Mexico City, Mexico) were also used.

### 2.2. Film Preparation

The methodology proposed by Fonseca-García, Jiménez-Regalado [11] was followed for producing biodegradable films. The polymers were used in various concentrations: starch 5% (*w/w*), chitosan 1% (*w/w*), and poloxamer (0%, 1%, 3%, and 5% *w/w* of biopolymers). The polymer solutions were mixed in a ratio of 60:20:20, and glycerol was added as a plasticizer at 25% (*w* of glycerol/*w* of total polymers). Fifty milliliters of each polymer dispersion was poured into a plastic mold and dried at 60 °C (Duo-Vac Oven, Lab-Line Instruments, Inc., Melrose Park, IL, USA) for four hours. The dried films were peeled off and stored in airtight storage bags for 16 months in the dark and at ambient conditions with a relative humidity (RH) of 32.73% and temperature of 25 °C.

### 2.3. Color

Color was determined using a portable spectrophotometer (Mini Scan EZ 4500L, HunterLab, Reston, VA, USA) based on the CIELab scale with the coordinates L*, a*, and b* [18]. The films were placed on a white copy paper used as standard with color coordinate values of L* = 91.76, a* = 2.14, and b* = −10.64.

### 2.4. Contact Angle

The contact angle was determined using the video goniometer (Rame-Hart instrument co., Succasunna, NJ, USA) to determine the hydrophobicity of the films [10]. A 5-µL drop of deionized water was placed on the opaquest side of a film sample (the side in contact with the mold) with dimensions of 3 cm × 1.5 cm, using a micropipette, and six measurements were recorded. Subsequently, with a program created using LabVIEW 2014 software, the image was captured, and angles formed by the water droplets on the film were calculated using ImageJ software.

### 2.5. Scanning Electron Microscopy

To analyze the morphology of the packaging films, SEM (JCM-6000, JEOL, Tokyo, Japan) was performed with a voltage of 10 kV. The films were coated with a gold-palladium layer (DESK II coating system, Denton Vacuum Inc., Moorestown, NJ, USA) [11].

### 2.6. Fourier Transform Infrared Spectroscopy (FTIR)

The vibrational modes of principal functional groups present in the packaging film were analyzed by FTIR spectrum using a Nicolet iS50 FTIR (Thermo Fisher Scientific, Madison, WI, USA) by ATR technique [10].

### 2.7. X-ray Diffraction (XRD) Analysis

The XRD of biodegradable films was determined using an X-ray diffractometer (D500, Siemens Aktiengesellschaft, Munich, Germany) with a voltage of 35 kV, current of 25 mA, and Cu Kα radiation of 1.5406 Å over a Bragg angular range (2θ) of 5–35° [11].

### 2.8. Differential Scanning Calorimetry

DSC was used to measure the gelatinization peak (*T_P_*), melting temperatures (*T_m_*_1_ and *T_m2_*), and the melting enthalpy (Δ*H_m_*_2_). The analyses were performed at a temperature range of 25–300 °C under a nitrogen atmosphere (50 cm^3^ min^−1^) using a thermogravimetric analyzer (DSC 2 STAR System, Mettler-Toledo AG, Schwerzenbach, Switzerland), according to the standard ASTM D3418-15 [19].

### 2.9. Water Solubility

To determine the susceptibility of biodegradable films in regards to contact with liquid water at room temperature, the methodology proposed by Fonseca-García, Jiménez-Regalado [11] was followed without modifications.

### 2.10. Mechanical Properties

The tensile strength and percentage of elongation at break were determined using the methodology proposed by Fonseca-García, Jiménez-Regalado [11]. The experimental data regarding the mechanical properties were analyzed using OriginPro 8.5.0 SR1 software (OriginLab Corporation, Northampton, MA, USA) through an analysis of variance and the Tukey test with a significance level of *p* < 0.05.

## 3. Results and Discussion

### 3.1. Appearance and Color

**Figure 1:**
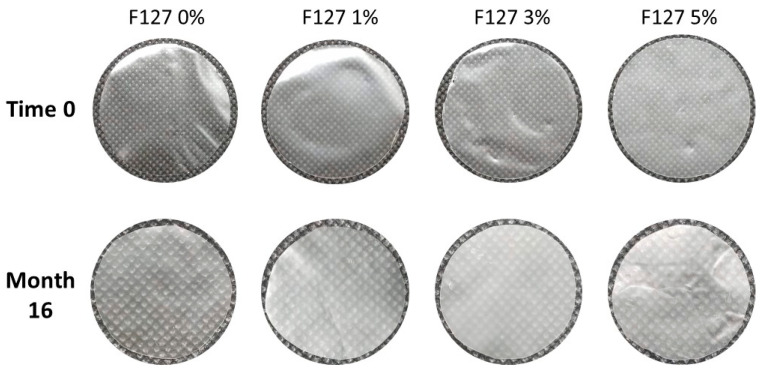
Biodegradable films based on corn starch and chitosan with pluronic F127 at 0%, 1%, 3%, and 5%; images were captured in the initial storage time (time 0) and after 16 months of storage at room temperature; the photos of the films at 16 months were taken at higher magnification.

All biodegradable films developed in this study appeared to be homogeneous. Figure 1 shows the starch-chitosan composite films with different proportions of the poloxamer pluronic F127 stored for 0 and 16 months; the images were captured on a black background for contrast. All films presented a translucent white color, as seen in Figure 1, and exhibited no significant visible changes during the 16-month storage process. They did not become brittle or more brittle to handle with time in storage. No visibly considerable color change was noted in the films when compared to the initial time (0) with the samples stored for 16 months. However, when evaluating the film color using a colorimeter, it was observed that the samples did change their coloration after 16 months of storage when compared to the initial time, as indicated by the values of the L*, a*, and b* color parameters in Table 1.

### 3.2. Morphology by SEM

**Figure 2:**
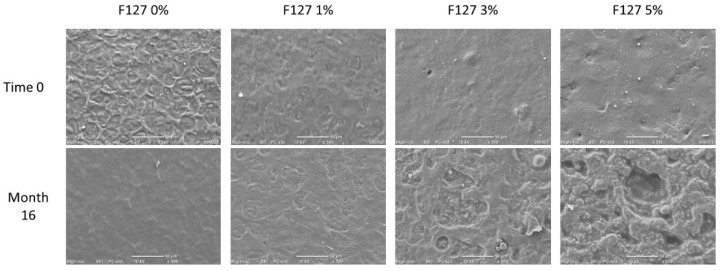
SEM images for the surface (500×) of composite films of corn starch-chitosan with pluronic F127 at ratios of 0%, 1%, 3%, and 5% stored for 0 and 16 months.

Figure 2 shows a comparison of the films with different amounts of poloxamer evaluated at time 0 and 16 months of storage. The evaluation of the initial time (zero) showed that in the biodegradable films formulated from starch and chitosan, the presence of the poloxamer significantly improved the morphology of the surface of these materials. In the film formulated without F127, an irregular surface was observed without cracks. This appearance was gradually reduced until a smoother surface was obtained with an increase in the content of F127.

A significant change was observed in the morphology of all the composite biodegradable films after 16 months of storage under ambient conditions; however, the most noticeable or drastic changes were observed in the materials containing F127 at concentrations of 3% and 5% (Figure 2). A less homogeneous morphology was observed in these films, which may be due to a change in the organizational structure of the starch chains. Garalde, Thipmanee [20] reported a similar morphology in thermoplastic starch/poly (butylene adipate-co-terephthalate) (PBAT) films, which occurred when the starch was selectively removed from the composite film.

### 3.3. Contact Angle

The wettability of the biodegradable films was evaluated in terms of the contact angle; Table 1 shows the contact angle values of the starch-chitosan-poloxamer (F127) films. In films F127 0% and F127 1%, after 16 months of storage, the hydrophilicity increased as the contact angle decreased from 99.93° to 67.66° and 33.22° to 31.89°, respectively. In the case of F127 3% and F127 5%, the hydrophilicity decreased as the contact angle increased from 43.88° to 46.09° and 46.25° to 50.74°, respectively. The results suggest that pluronic F127 has a considerable effect on the wettability of biodegradable starch-chitosan films.

### 3.4. FTIR

**Figure 3:**
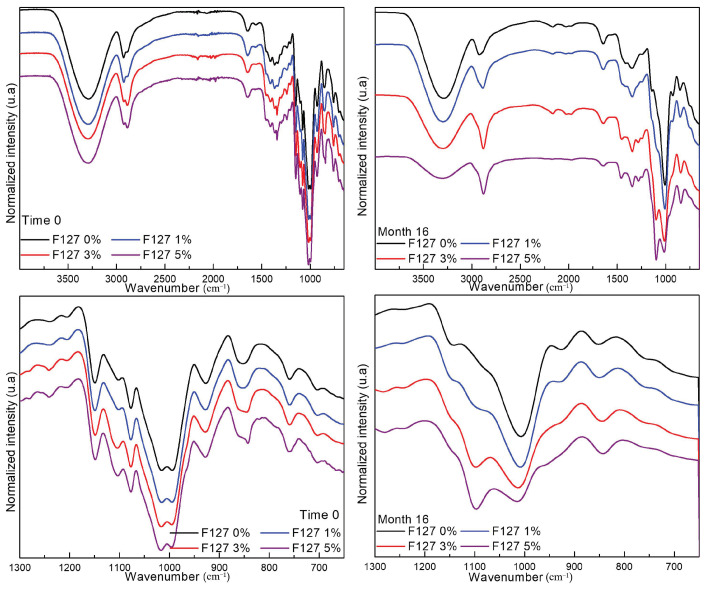
FTIR spectra of biodegradable films of corn starch-chitosan with pluronic F127 at ratios of 0%, 1%, 3%, and 5% stored for 0 and 16 months.

Figure 3 shows the spectra of the films stored for different durations. After 16 months of storage, the FTIR-ATR analysis showed modifications in the absorption in the region of 1300 cm^−1^ to 900 cm^−1^ for all materials (F127 0%, F127 1%, F127 3%, and F127 5%); the bands in this region are sensitive to the gelation of starch because of their association with C–O stretching of the ring, linkages (C–O–C), and COH groups. Notably, the band at 1017 cm^−1^ is reported as sensitive to amorphous starch, which is constant in the spectra after 16 months of storage [21,22,23]. Casu and Reggiani [24] reported that the band at 3300 cm^−1^, attenuated after 16 months of storage, can be assigned to the O-H stretching of the groups in amorphous amylose. Moreover, the molecule of water showed that the absorption bands at 3300 and 1646 cm^−1^ are associated with OH stretching and deformation vibrations, respectively [24]. In addition, the absorption band at 2883 cm^−1^ was associated with C-H bond stretching, which did not show a modification in the four materials after 16 months [25]. Finally, another important phenomenon identified in the spectra of all films after storage was the disappearing of the absorption band at 1150 cm^−1^, which was associated with C-O stretching of C–O–C in glycosidic linkage; this result suggests the depolymerization of the starch molecule [26].

### 3.5. X-ray Diffraction

**Figure 4:**
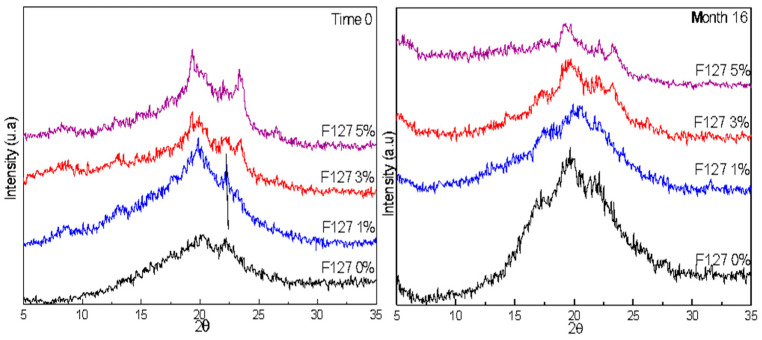
X-ray diffraction patterns of biodegradable films of corn starch-chitosan with pluronic F127 at ratios of 0%, 1%, 3%, and 5% stored for 0 and 16 months.

To determine if there was retrogradation or modification in the conformation of the structural matrix of the biodegradable films, Figure 4 shows the XRD patterns of biodegradable films F127 0%, F127 1%, F127 3%, and F127 5%. The patterns show that there was a higher atomic ordering in the biodegradable film in F127 0% after 16 months of storage. Peaks at 16°, 19.65°, and 22° were defined after storage; these peaks were related to the crystalline starch, which indicates the retrogradation of starch in this film. However, in films F127 1%, F127 3%, and F127 5%, there was no increase in the atomic ordering after 16 months of storage, as peaks related to starch could not be identified. In F127 3% and F127 5% films, there were two peaks at 19° and 23°, which were less intense after 16 months; these peaks were associated with pluronic 127 [11]. The X-ray patterns indicate that pluronic F127 avoided retrogradation after the storage period of 16 months. Mina Hernandez [27] reported a significant increase in the rearrangement of the polymer chains of mixtures of thermoplastic starch and polycaprolactone during short storage periods (5 and 26 days). Furthermore, the author found that the retrogradation of starch-based polymers occurs more rapidly when the materials are stored in conditions of high RH, which also negatively impacts their mechanical performance.

### 3.6. Thermal Behavior by DSC

The results of the thermal behavior of the biodegradable films are presented in Table 2. DSC experiments have shown that the melting temperature (*T_m_*) of the neat corn starch film decreases with a significant increase in enthalpy (∆*H_m_*), indicating a gain in the ordering of the polymeric structures involved. In contrast, chitosan films exhibited an increase of 15 °C after 16 months of storage. The enthalpy values for this biopolymer decreased as a function of the evaluated time. Notably, the mixture of starch and chitosan (F127 0%) exhibited a thermal behavior in which starch transitions predominated. The incorporation of the poloxamer in the starch-chitosan matrix ensured gelatinization, with the absence of the band at ~62 °C (*T_P_*, gelatinization peak). A decrease was observed in the melting temperature (56.1 °C) corresponding to neat F127. The increase in poloxamer content resulted in an increase in the melting temperature and a decrease in enthalpy after 16 months, with respect to its nonfunctionalized homologous (F127 0%). This allowed for demonstrating the plasticizing effect generated by the poloxamer due to the destabilization of the crystalline regions. The fusion of intra- and inter-molecular double helices and the partial recovery of the crystalline structure of amylopectin (Figure S1, Supplementary Material) can be observed [28]. The crystals formed during the starch retrogradation process are less orderly and homogeneous than native starch; this is reflected in their lower melting temperatures. This effect is similar to that observed in other starches, such as sago [29]. The DSC parameters of starch in biodegradable films are summarized in Table 2, and this result is in accordance with that obtained from the XRD analysis.

### 3.7. Water Solubility (WS)

The WS of biodegradable materials is a crucial parameter because it is important that they can decompose in both terrestrial and aquatic environments after being discarded, where they can potentially contaminate the environment on several occasions. A significant increase in the water solubility capacity of the starch-chitosan-based materials was observed (Table 1) at all poloxamer contents (1%, 3%, and 5%) after 16 months of storage with water solubility values of 18%, 24%, and 57%, respectively, compared to the materials evaluated at time zero [11]. Shaker, Elbadawy [30] found that by using different types of poloxamers, such as pluronic F127 and F68, the degree of solubility and the dissolution rate of drugs improved, thus facilitating their usage in pharmaceutical production.

### 3.8. Mechanical Properties

The mechanical performance of corn starch and chitosan films with various poloxamer concentrations was significantly affected with storage time, as shown in Table 1. A slight increase in the stiffness of the materials was observed, presenting an increase in the tensile stress values. In comparison, the percentage of elongation of the materials decreased when the content of poloxamer was >3%. This mechanical behavior may be associated with two changes in the films. First, SEM micrographs showed that when the poloxamer content was >3% in the films, a higher superficial modification was observed. Second, based on the FTIR spectra of the films, the starch contained in these materials was presumed to depolymerize after storage for 16 months. Furthermore, hydrogen bonds are the predominant ones in these biodegradable films; however, this type of bond is known to be weak, and the tenacity is affected in this case. This mechanical property is diminished by lower values of elongation, without resistance being sacrificed.

## 4. Conclusions

Storage time can significantly modify the physicochemical, morphological, and structural properties of biodegradable films comprising starch, chitosan, and poloxamer. This information is important to ascertain the state of packaging materials of biodegradable origin when stored at room temperature. Based on physicochemical characterization, there is evidence suggesting that poloxamer avoids the retrogradation of cornstarch. The biodegradable films showed a slight modification in their surface morphology after 16 months, similar to erosion, and this phenomenon can modify the color, WS, mechanical behavior, and wettability of films. Lower melting temperatures and enthalpies in the poloxamer films, as well as storage for 16 months, indicate that the retrogradation of amylopectin only partially recovers the crystalline structure of the native starch. The use in the pharmaceutical, medical, and packaging industry of a biodegradable material such as the one developed in this study may be possible, owing to the favorable properties observed in its mechanical performance and acceptable solubility in liquid water, which can be sufficiently solubilized in a short period of time with the presence of the poloxamer.

## Figures and Tables

**Table 1:** Effect of poloxamer content and storage time on water solubility, color, contact angle, and mechanical properties of corn starch-chitosan biodegradable films.

Content of F127	Water Solubility (%S)	Tensile Strength (MPa)	Elongation at Break (%)	Hunter Color Values ^1^			Contact Angle (°)
				L*	a*	b*	Ѳ
Time 0 months							
F127 0%	42.58 ^§^	4.24 ^a,^^§^	149.05 ^c,^^§^	93.74	2.29	−11.50	99.93
F127 1%	7.81 ^§^	6.49 ^a,b,^^§^	53.30 ^a,^^§^	93.52	1.87	−9.31	33.22
F127 3%	4.02 ^§^	4.96 ^a,^^§^	95.20 ^b,^^§^	94.22	2.31	−11.56	43.88
F127 5%	3.34 ^§^	3.68 ^a,^^§^	84.96 ^b,^^§^	94.53	2.10	−10.54	46.35
Time 16 months							
F127 0%	24.40	5.18 ^b^	68.53 ^c^	90.88	1.53	−8.05	67.66
F127 1%	17.66	7.01 ^c^	60.60 ^c^	90.81	1.47	−7.75	31.89
F127 3%	24.17	5.96 ^b^	30.67 ^b^	91.09	1.41	−7.79	46.09
F127 5%	56.98	3.27 ^a^	12.40 ^a^	89.53	1.30	−6.02	50.74

Mean values of at least three replicates. ^§^ Experimental data and significant difference taken from Fonseca-García, Jiménez-Regalado [11]. ^1^ Hunter color parameters, L*: color coordinate, lightness (0 = black, 100 = white); a*: color coordinate, greenness and redness (+ = red, − = green); b*: color coordinate, blueness to yellowness (+ = yellow, − = blue). L* standard= 91.76, a* standard = 2.14, and b* standard = −10.64. ^a,b,c^: values with different letter in a same column denote significant difference.

**Table 2:** DSC parameters of biodegradable films of corn starch-chitosan with pluronic F127 at ratios of 0%, 1%, 3%, and 5% stored for 0 and 16 months.

Film Sample	T_p_ (°C)	T_m1_ (°C)		T_m2_ (°C)		∆H_m2_ (J g^−1^)	
	Month 0	Month 0	Month 16	Month 0	Month 16	Month 0	Month 16
Corn starch	63.13	--	--	141.03	117.75	6.0	18.7
Chitosan	--	--	--	104.68	119.47	28.0	19.6
F127		56.10	56.10				
F127 0%	62.06	--	--	138.96	106.63	6.4	10.3
F127 1%	--	45.52	51.52	137.63	112.94	8.4	9.1
F127 3%	--	51.95	51.68	--	108.05	--	6.3
F127 5%	--	47.84	51.46	114.36	115.22	8.5	4.0

-- negligible.

## Data Availability

The data presented in this study are available on request from the corresponding author.

## Supplementary Materials

The following are available online at https://www.mdpi.com/article/10.3390/polym13142341/s1, Figure S1: DSC thermograms of biodegradable films of corn starch-chitosan with pluronic F127 at ratios of 0%, 1%, 3%, and 5% and stored for 0 and 16 months.
