# Supplementary material for: Effects of Poloxamer Content and Storage Time of Biodegradable Starch-Chitosan Films on Its Thermal, Structural, Mechanical, and Morphological Properties
Source: Polymers (Basel). 2021 Jul 17;13(14):2341. doi: 10.3390/polym13142341 (PMC8309472; doi:10.3390/polym13142341)
Supplement: Supplementary file 1 [file polymers-13-02341-s001.zip › polymers-1292822-supplementary.pdf]

polymers

MDPI

Supplementary Material: Effect of Poloxamer Content and Storage Time of Biodegradable Starch-chitosan Films: Thermal, Structural, Mechanical, and Morphological Properties

Abril Fonseca-García, Carolina Caicedo, Enrique Javier Jiménez-Regalado, Graciela Morales, Rocio Yaneli Aguirre-Loredo

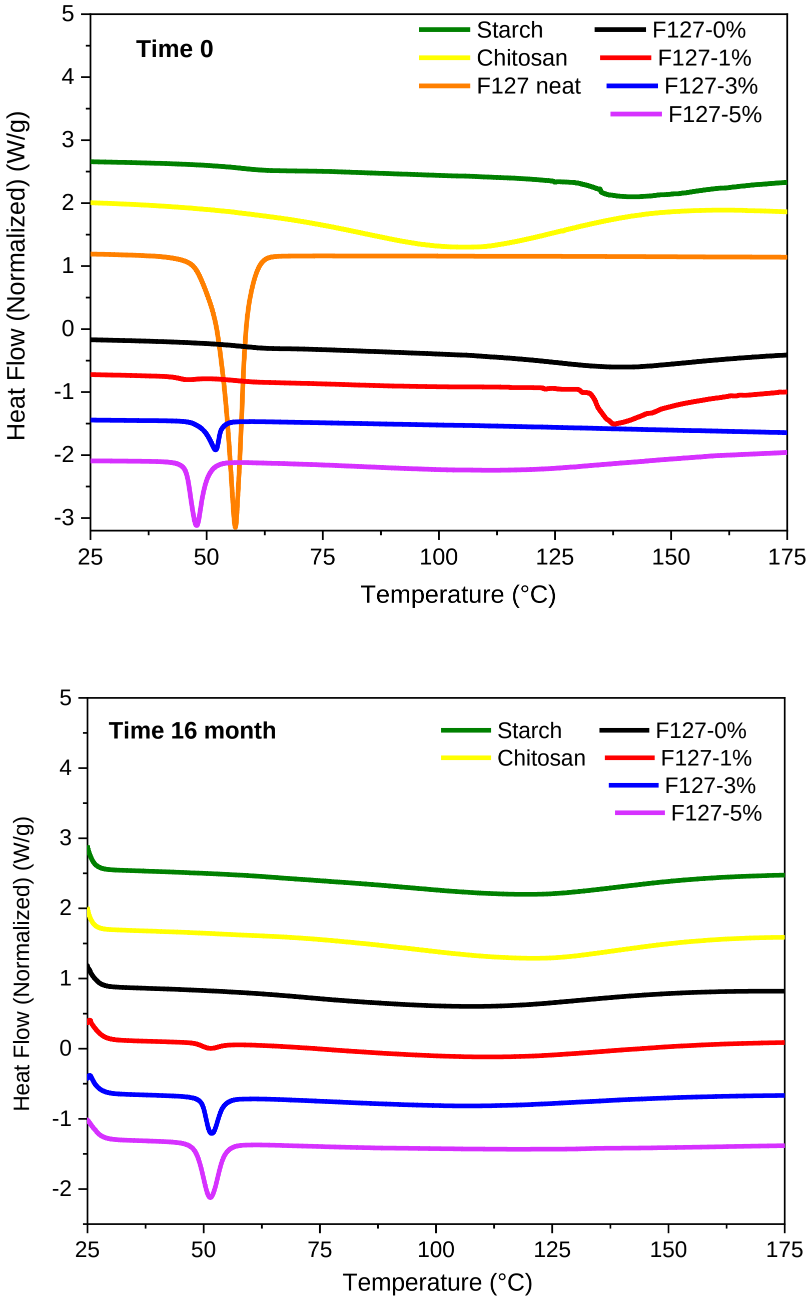

Figure S1. DSC thermograms of biodegradable films of corn starch-chitosan with pluronic F127 at ratios of 0, 1, 3, and 5% stored at 0 and 16 months.
